# Evaluating the effects of increasing physical activity to optimize rehabilitation outcomes in hospitalized older adults (MOVE Trial): study protocol for a randomized controlled trial

**DOI:** 10.1186/s13063-014-0531-y

**Published:** 2015-01-15

**Authors:** Catherine M Said, Meg E Morris, Jennifer L McGinley, Cassandra Szoeke, Barbara Workman, Danny Liew, Keith Hill, Michael Woodward, Joanne E Wittwer, Leonid Churilov, Cameron Ventura, Julie Bernhardt

**Affiliations:** Department of Physiotherapy, School of Allied Health, La Trobe University, Kingsbury Drive, Bundoora, Victoria 3086 Australia; Department of Physiotherapy, Austin Health, 145 Studley Rd, Heidelberg, Victoria 3084 Australia; Department of Physiotherapy, Melbourne School of Health Sciences, The University of Melbourne, Parkville, Victoria 3010 Australia; Department of Psychiatry & National Ageing Research Institute, The University of Melbourne, Parkville, Victoria 3050 Australia; Rehabilitation and Aged Services, Monash Health, Warrigal Rd, Cheltenham, Victoria 3192 Australia; Monash Ageing Research Centre (MONARC), Monash University, Warrigal Rd, Cheltenham, Victoria 3192 Australia; Melbourne EpiCentre, The University of Melbourne and Melbourne Health, Parkville, Victoria 3050 Australia; School of Physiotherapy and Exercise Science, Curtin University, Kent St, Bentley, Western Australia 6102 Australia; Aged Care Services, Austin Health, 300 Waterdale Rd, Heidelberg West, Victoria 3081 Australia; Department of Medicine, The University of Melbourne, Parkville, Victoria 3010 Australia; Statistics and Decision Analysis Academic Platform, The Florey Institute of Neuroscience & Mental Health, 245 Burgundy St, Heidelberg, Victoria 3084 Australia; Stroke Division, The Florey Institute of Neuroscience and Mental Health, 245 Burgundy St, Heidelberg, Victoria 3084 Australia

**Keywords:** mobility limitation, rehabilitation, exercise therapy, hospitalization, randomized controlled trial

## Abstract

**Background:**

Older adults who have received inpatient rehabilitation often have significant mobility disability at discharge. Physical activity levels in rehabilitation are also low. It is hypothesized that providing increased physical activity to older people receiving hospital-based rehabilitation will lead to better mobility outcomes at discharge.

**Methods/Design:**

A single blind, parallel-group, multisite randomized controlled trial with blinded assessment of outcome and intention-to-treat analysis. The cost effectiveness of the intervention will also be examined. Older people (age >60 years) undergoing inpatient rehabilitation to improve mobility will be recruited from geriatric rehabilitation units at two Australian hospitals. A computer-generated blocked stratified randomization sequence will be used to assign 198 participants in a 1:1 ratio to either an ‘enhanced physical activity’ (intervention) group or a ‘usual care plus’ (control) group for the duration of their inpatient stay. Participants will receive usual care and either spend time each week performing additional physical activities such as standing or walking (intervention group) or performing an equal amount of social activities that have minimal impact on mobility such as card and board games (control group). Self-selected gait speed will be measured using a 6-meter walk test at discharge (primary outcome) and 6 months follow-up (secondary outcome). The study is powered to detect a 0.1 m/sec increase in self-selected gait speed in the intervention group at discharge. Additional measures of mobility (Timed Up and Go, De Morton Mobility Index), function (Functional Independence Measure) and quality of life will be obtained as secondary outcomes at discharge and tertiary outcomes at 6 months follow-up. The trial commenced recruitment on 28 January 2014.

**Discussion:**

This study will evaluate the efficacy and cost effectiveness of increasing physical activity in older people during inpatient rehabilitation. These results will assist in the development of evidenced-based rehabilitation programs for this population.

**Trial registration:**

Australian New Zealand Clinical Trials Registry ACTRN12613000884707 (Date of registration 08 August 2013); ClinicalTrials.gov Identifier NCT01910740 (Date of registration 22 July 2013).

## Background

The aging population represents a significant and growing challenge to the Australian [1], US [2] and other healthcare systems [3]. In Australia in 2011 and 2012, people aged over 65 years accounted for 48% of all hospital bed days and 39% of hospital discharges [4], despite only representing 14.4% of the population [1]. Older people often require inpatient mobility rehabilitation after discharge from an acute hospital to enable them ‘to reach and maintain their optimal physical, sensory, intellectual, psychological and social functional levels’ [5]. In turn, this can help them to return to previous living arrangements and lifestyle.

Mobility outcomes following rehabilitation are often poor in older adults. Many older people are frail and have impaired mobility on admission to an inpatient geriatric rehabilitation unit, and evidence indicates that mobility outcomes remain suboptimal in many older people following discharge from these facilities [6,7]. For example, a recent study found that 14% of older people discharged from an inpatient geriatric rehabilitation unit were unable to walk 10 meters [6]. Of those who could walk 10 meters, only 31% were independent when walking up stairs. Gait speed was also reduced, with a very low median speed of 0.46 m/sec (IQR = 0.32), compared to a mean speed of 1.2 to 1.3 m/sec in healthy older adults [6]. Mobility problems commonly persist following discharge, with only 41% of older adults being able to walk 800 meters or climb a flight of stairs 3 months after discharge [7]. Poor mobility can have serious consequences for older adults. It is associated with the need for long-term care [8], loss of functional independence and mortality [9] and falls [10]. It is therefore important to maximize recovery of mobility in older people with mobility disability.

While it is widely believed that bed rest and inactivity in hospital are detrimental for mobility and function [11], there are no definitive clinical guidelines on optimum physical activity levels for older adults undergoing inpatient rehabilitation. Despite the publication of physical activity guidelines for older people [12-15], a gap exists in guidelines for frail older people or those who have multiple chronic diseases and are undergoing rehabilitation. A number of studies indicate that activity levels in rehabilitation are low [16-18] and that physical activity levels in aged care rehabilitation are particularly low [19]. This raises the possibility that better outcomes could be achieved by adding interventions, strategies and systems to increase physical activity. Increasing physical activity is a simple intervention likely to have a positive impact on recovery of mobility.

Surprisingly, there is limited research examining the effect of increasing physical activity on mobility outcomes in hospitalized older people undergoing rehabilitation. These patients typically have a wide variety of admission diagnoses and multiple co-morbidities, which may include cognitive impairment [6]. As a consequence, they are often excluded from clinical trials. A recent systematic review [20] found providing hospitalized patients with an additional average of 19 minutes physiotherapy per day led to improvements in mobility and quality of life and reductions in length of stay. However, the average age of participants in the review was only 69.8 years. There is some evidence that a multidisciplinary exercise intervention in an acute hospital reduces acute length of stay and increases the proportion of older people who return home [21]. There is also promising evidence that increasing physical activity in a range of outpatient, home and residential care settings can improve outcomes in some older people [22-24]. A pilot study conducted by our group demonstrated the feasibility of utilizing a randomized controlled trial (RCT) to examine the impact of increased physical activity on mobility outcomes in older people receiving inpatient geriatric rehabilitation. A satisfactory recruitment and retention rate was achieved, protocols to increase physical activity were developed, increases in physical activity were obtainable, there was no evidence of group contamination, and there was no increase in adverse events [25]. Given the success of the pilot trial and the potential for mobility improvements via increasing physical activity, a larger and appropriately powered RCT to further explore the efficacy of increasing physical activity in older people on mobility and other outcomes is warranted.

The proposed study will test the primary hypothesis that increasing physical activity in older people during rehabilitation, compared with a control group, will lead to better mobility at discharge, as measured by self-selected gait speed. Secondary hypotheses follow concerning the increase of physical activity during rehabilitation:

1. It will lead to significantly greater improvements in secondary measures of mobility and function both at discharge and 6 months after discharge.
2. It will lead to a better quality of life 6 months after hospital discharge.
3. It will be cost effective compared to usual care.

## Methods/Design

### Design

**Figure 1:**
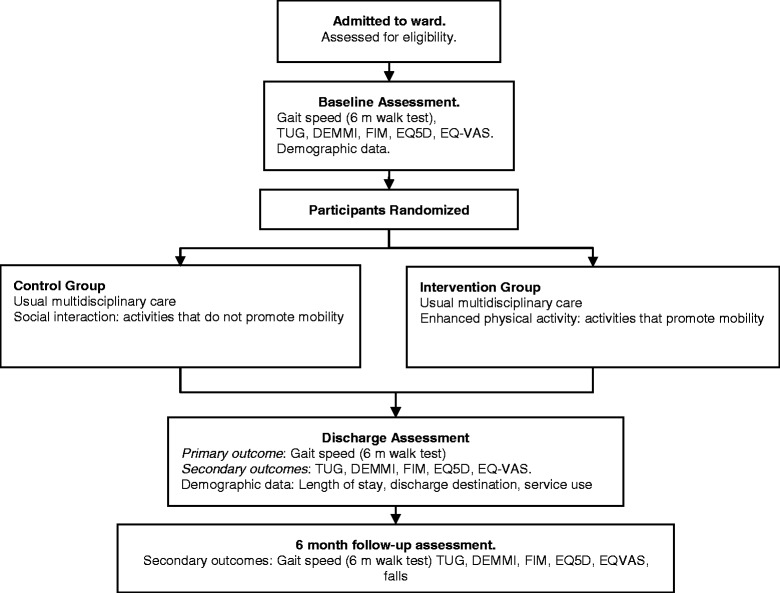
**Participant flow-through study.** TUG, Timed up and go; DEMMI, De Morton Mobility Index; FIM, Functional Independence Measure; EQ5D, Euro QoL Health Questionnaire; EQ-VAS, Euro QoL, Visual Analog Scale.

An investigator and assessor-blinded, parallel group multisite RCT will be used to compare enhanced physical activity to ‘usual care plus’ provided to older people receiving in-hospital geriatric rehabilitation at hospitals in the Melbourne metropolitan area of Victoria, Australia. Figure 1 provides an overview of the study design. Reporting of results will conform to the recommendations of the Consolidated Standards of Reporting Trials (CONSORT) statement [26]. Ethical approval has been obtained from the La Trobe University Human Ethics Committee (12-122), and from ethics committees at each site prior to commencement at that site (Austin Health Human Research Ethics Committee approval H2013/05042, Monash Health Human Research Ethics Committee approval14117X). The trial has also been registered with The University of Melbourne (Ethics ID 1340834) and Curtin University (HR 25/2014). Any protocol amendments will be submitted to the relevant Ethics Committees for review and will be updated on the trial registries.

### Participants

Older people will be recruited within 48 hours of admission to rehabilitation and geriatric evaluation and management wards of the participating hospitals. People admitted to these wards are medically stable, but may have chronic or complex health conditions that require treatment and management by a multidisciplinary team, or require a period of rehabilitation to maximize function. A member of the research team will screen each person upon admission and approach potential participants in person. To be included in the study, participants must be aged 60 years or older and have a goal of admission to ‘improve mobility or improve walking’ as determined by admission referral or the treating therapist. Participants will be excluded if (1) there are specific medical restrictions that limit mobilization, (2) mobility goals are limited to non-weight bearing mobility goals, (3) they are already enrolled in an RCT, or (4) the primary reason for admission is to await residential care or carer training. Informed consent will be obtained from the participant, or ‘responsible person’ if the participant is unable to give consent due to cognitive impairment. Interpreters will enable recruitment of people who are non-English speaking.

### Randomization

As study site and mobility on admission are potential confounders, randomization will be blocked and stratified by site and mobility level. At baseline, the mobility of participants will be classified as either non-ambulant (Level 1 or 2 as detailed in Table 1) or ambulant (Level 3 or 4 as detailed in Table 1)*.* This classification will be used for stratification by mobility level. Once consent has been obtained and baseline data collected, participants will be individually randomized to an intervention group (enhanced physical activity) or a control group (usual care plus) according to a computer generated randomization procedure performed by a third party. Group assignment will only be available to intervention staff and the project manager, and will be accessed online via a password protected site.

**Table 1:** **Functional classification of participants and summary of activities for intervention group**

**Level**	**Function**	**Intervention**
1	Patient is unable to transfer out of bed without maximum assistance (two persons or a hoist) and has poor static and dynamic sitting balance (unable to sit independently).	Bed exercise program (including lower limb, upper limb and abdominal strength and bed mobility) and sitting balance exercises.
2	Patient can transfer out of bed with assistance from one person, has independent sitting balance, but is unable to stand independently. Requires moderate assistance from two people to walk.	Sitting exercise program including targeted lower limb strengthening exercises. Sit to stand exercises, standing balance exercises, stepping/marching on the spot as able (using rails/ gait aids for safety as indicated). Activities from the previous level may be included if specifically indicated. For example, if the participant is unable to perform full range movement against the effects of gravity, specific lower limb muscle strengthening exercises may be performed on the bed.
3	Patient can walk with minimal assistance from one person.	Walking exercises, sit to stand exercises, standing balance exercises, and step up exercises. Targeted lower limb strength exercises (where possible closed chain or functional strengthening exercises).
4	Supervision only or independence with ambulation. Requires minimal assistance or supervision on stairs.	Stairs exercises, walking exercises (including outdoor mobility), step up exercises, standing balance exercises. Targeted lower limb strength exercises as indicated (where possible closed chain or functional strengthening exercises).

Reprinted from Said, C. M., Morris, M. E., Woodward, M., Churilov, L., & Bernhardt, J. (2012). Mobility in older adults receiving hospital based rehabilitation: A phase II feasibility study. BMC Geriatrics, 12:26. doi: 10.1186/1471-2318-12-26.

### Outcome measures

#### Primary outcome

The primary outcome measure is mobility on discharge from hospital as assessed using gait speed. This will be measured using the 6-meter walk test [27] at self-selected speed by a trained assessor blinded to group allocation. Participants will be instructed to walk at self-selected speed along a 10-meter walkway, and a stopwatch will be used to measure speed over the middle 6 meters. Participants will be allowed to use a gait-aid if required, and this will be recorded. Up to three trials will be performed, and the best performance recorded. Participants unable to complete the test will be given a score of 0 m/sec. Gait speed was chosen as a primary outcome measure as it is associated with the need for long-term care [8], functional dependence, mortality [9] and falls [10]. Gait speed can also predict activity levels at home and in the community [28,29] and increases in gait speed are associated with improvements in overall health status [30]. Furthermore, gait speed has good retest reliability [27,31] and is responsive to changes in walking ability [32-34].

#### Secondary outcomes

Secondary outcome measures include mobility on discharge from hospital as assessed using the Timed Up and Go (TUG) [31,35] and the De Morton Mobility Index (DEMMI) [36-38]. Gait speed will also be measured 6 months postdischarge as a secondary outcome measure. Additional secondary outcomes include function, as measured using the Functional Independence Measure (FIM^TM^) [39], health-related quality of life, as measured by the EuroQol health questionnaire (EQ5D) and the EuroQol Visual Analog Scale (EQ-VAS) [40] and subacute length of stay (LOS). These tests will be administered in accordance with previously published protocols. If a participant is unable to complete the EQ5D or EQ-VAS due to cognitive impairment, a proxy will complete it on their behalf.

#### Tertiary outcomes

Tertiary outcomes of mobility, function and quality of life will be obtained 6 months postdischarge, using the TUG [31,35], the DEMMI [36-38], the FIM [39], the EQ5D and the EQ-VAS.

Falls will be monitored throughout the study. Falls will be defined as ‘an unexpected event in which you come to rest on the ground, floor or lower level’ [41]. Data on falls during the period of hospitalization will be obtained via review of medical records and hospital incident reports. Falls in the 6 months postdischarge will be recorded prospectively via a monthly falls calendar provided to participants at discharge. Participants will be asked to record the date of any falls and whether or not advice or treatment from a health professional had been sought related to the fall. They will be asked to return the calendar at the end of each month, and will receive a telephone reminder if the falls calendar is not returned. This method of falls data collection is considered the ‘gold standard’ for collecting falls data [41].

### Assessments

Each assessment will be completed at three time points by an assessor blinded to group allocation. All assessors will be trained to ensure the assessments are standardized and will be FIM^TM^ credentialed.

#### Baseline

The baseline assessment will be completed within 48 hours of admission. In addition to primary and secondary outcome measures collected at baseline, additional demographic and clinical data will be obtained. These data will include the participant’s age, sex, height and weight, acute LOS, mental state (measured by the Mini Mental State Examination [42]), frailty (measured using the modified Fried Frailty Index) [43,44], co-morbidities (age adjusted Charlson Comorbidity Score [45]), depression (Geriatric Depression Scale [46]), medications, self (or carer) reported falls in the last 12 months and social situation.

#### Discharge

The second assessment will be completed within 48 hours of discharge. Self-selected gait speed (primary outcome) at this assessment will be the primary endpoint of the study. In addition to the primary and secondary outcome assessments, discharge destination and service referrals on discharge will also be collected. For participants discharged to residential care, the day the treating team determined that they required residential care placement and required paperwork was completed will be used in lieu of actual discharge date for this analysis.

#### Six months postdischarge

The third and final assessment will be conducted 6 months postdischarge in the person’s residence. In addition to further secondary and tertiary outcome assessments, falls data and any associated health professional advice or treatment will be collected.

### Interventions

Both the intervention and control group will be provided with usual care, which will include ongoing therapy provided by a multidisciplinary team including nurses, medical staff, physiotherapists, occupational therapists and other allied health professionals as required throughout their in-patient rehabilitation. Both groups will receive additional therapy sessions, but the content of these sessions will differ depending on group allocation.

#### Intervention group: enhanced physical activity

In addition to usual care, participants in the enhanced physical activity intervention group will undertake 1 to 2 sessions of additional physical activities on weekdays, and a further two sessions each weekend throughout their inpatient stay (anticipated average of 19 days, based on data from the participating hospitals). Programs will be individually tailored with the aim of increasing the amount of time participants spent performing mobility exercises, such as standing or walking. Intervention protocol guides, which were trialed in the pilot study, [25] will be specified for participants according to their functional level (summary provided in Table 1; full details provided in trial manual available from authors). Specific targets for activity levels on weekdays and weekends have been set, based on pilot data [19,25]. All sessions will be provided by a physiotherapist or physiotherapy assistant not involved in the provision of usual care to participants. At the end of each intervention session, the physiotherapist or allied health assistant will record the amount of time spent performing each activity in 5-minute increments. No specific program to be completed outside of these sessions will be provided.

#### Control group

To control for the extra hours of social interaction received by participants in the enhanced physical activity group, participants in the control group will undertake additional seated or resting social activities including card and board games, conversation and reading, as well as upper limb and other physical exercises that have minimal impact on mobility. These sessions will be provided by either a physiotherapist or physiotherapy assistant, and time involved in these sessions will be recorded.

### Blinding, contamination and monitoring

All assessments will be performed by clinicians blinded to group allocation. All investigators, with the exception of the project manager (JW) will remain blinded to group assignment. Staff providing the intervention cannot be blinded. To minimize the risk of usual-care staff altering practice during the period of data collection, staff not directly involved in the research will not be told the specific aims of the study. As it is essential that activity delivered as part of the intervention is in addition to usual care, intervention staff will not be involved in other aspects of patient care. To monitor usual-care activities, usual-care physiotherapy staff will record the amount of time performing various activities in physiotherapy sessions for all participants in five minute increments. To ensure the correct ‘dosage’ of intervention activity is delivered, time spent performing upright activities will be monitored, and feedback on dose will be provided to intervention therapists by the project manager. To monitor for activity levels and contamination throughout the day, participants in both study groups will have their activity levels monitored over a 5-day period using a SenseWear armband [47-49]. Regular monitoring of activity will help identify deviations from protocol and potential contamination of usual care. These data will be reviewed monthly by the Project Management Committee and strategies to address any issues implemented.

### Risk management and safety monitoring

There is a risk that the intervention may result in an increase in adverse events, although no differences between groups were reported in the pilot study [25]. In the event of a fall or medical event, standard hospital procedures will be followed. Falls during hospital stay, mortality and unplanned re-admissions to an acute service (during rehabilitation or within six months postdischarge) will be recorded on an incident form, monitored by an independent Data Safety and Monitoring Committee and reported to the relevant ethics committees.

### Data management

All data will be stored in a confidential manner. Participants will be assigned a code, which will be used for all data management and analyses. Paper data will be stored at the local site in a locked filing cabinet. Coded electronic data will be stored on a computer server at The Florey Institute of Neuroscience and Mental Health for the duration of the study. Access to the computer files will be password-protected and accessible only to the research team. Data quality will be monitored by the project manager on a regular basis and reported to the management committee. The project manager will also conduct regular site visits to monitor trial conduct. All project investigators will have access to the final, de-identified data set.

### Sample size estimation

The sample size for this study was calculated based on pilot data, which showed that the median gait speed of people discharged from aged care rehabilitation was 0.46 m/sec (range 0.1 to 1.2 m/sec) [6], which allowed a mean gait speed (0.46 m/sec) and variance (0.18 m/sec) to be estimated [50]. Sample size estimation was based on the intervention group achieving an increase in gait speed of at least 0.1 m/sec compared with the control group, the recommended minimal clinically important difference for gait speed in older adults [30,51]. With a two-tailed significance threshold alpha of 0.05 and power to yield a statistically significant result set at 90%, a sample size of 69 participants in each group is required. To account for an estimated 25% dropout rate and 14% of participants being unable to complete the walk test on discharge, as observed in pilot data [6,25], an estimated 198 participants will be required in total [52]. A recruitment rate of 34% was achieved in the pilot study. Based on average hospital admissions to the selected sites in this trial, and using a conservative recruitment rate of 20%, it is anticipated that the recruitment will be completed over 13 months.

### Statistical analysis

The primary analysis will be conducted on an intention-to-treat basis using data from all randomized participants, although a ‘per protocol’ analysis of the primary outcome will also be reported. Primary outcome values missing due to participants’ death will be imputed as zero. An independent adjudication panel, blinded to group assignment, will review records for participants’ who were discharged to an acute facility as medically unwell. Based on the reasons for transfer, the adjudication panel will determine whether the participant was likely to be able to walk and complete a 6-meter walk test at the time of transfer. If the adjudication panel determines the person was unlikely to be able to walk, the missing value will be imputed as zero. If the adjudication panel determines the person was likely to be able to walk, the last measure will be carried forward. Following that, multiple imputation of other missing primary outcome values will be conducted subject to the satisfaction of relevant missingness assumptions. Baseline data will be examined to ensure that the intervention and control groups are comparable using descriptive statistics with 95% confidence intervals.

All outcomes and analyses have been prospectively categorized as primary, secondary, or tertiary. Differences in both primary and secondary endpoints between the two arms of the study will be tested independently at the 0.05 level of significance. No formal adjustments will be undertaken to constrain the overall type I error associated with the secondary analyses. Their purpose is to supplement evidence from the confirmatory primary analysis to help more fully characterize the treatment effect. Results from the secondary analyses will be interpreted in this context.

#### Primary outcome analysis

Between-group differences in the primary outcome measure, self-selected gait speed on discharge, will be assessed using a one-way ANCOVA model that will include self-selected gait speed at baseline as a covariate.

#### Secondary outcomes analyses

Between-group differences in gait speed 6 months postdischarge will be assessed using a one-way ANCOVA model that will include self-selected gait speed at baseline as a covariate;

Between-group differences in TUG on discharge will be assessed using a Cox regression model that will include self-selected gait speed at baseline as a covariate;

Between-group differences in DEMMI score on discharge will be assessed using a one-way ANCOVA model that will include DEMMI score at baseline as a covariate;

Between-group differences in FIM score on discharge will be assessed using a median regression model that will include the FIM score at baseline as a covariate;

Between-group differences in EQ5D score on discharge will be assessed using a median regression model that will include the EQ5D score at baseline as a covariate;

Between-group differences in subacute LOS will be assessed using a median regression model that will include the FIM score at baseline as a covariate.

#### Exploratory analyses of tertiary endpoints

Between-group differences in the outcomes at 6 months postdischarge will be assessed using appropriate regression models that will include relevant measures at baseline as covariates. The exploratory longitudinal analysis for all outcomes over the period from baseline until 6 months postdischarge will be conducted using a mixed model repeated measures approach subject to the satisfaction of the underlying model assumptions.

### Health economic analysis

Health economic modelling will be undertaken to estimate the potential cost-effectiveness of the intervention arm compared to the control arm. Decision analysis [53] will be used to compare downstream consequences of enhanced physical care and Markov [54] and life-tabling [55] techniques will be applied to model outcomes beyond one year. The main output of interest will be incremental cost-effectiveness ratios in terms of net costs per unit of health gain. Net costs will comprise the costs of the enhanced physical activity program minus costs saved from the reduction in downstream health services utilization. Data on health services utilization will include clinical costing data provided by the hospitals, as well as data from the Australian Medical Benefits Scheme (MBS) and Pharmaceutical Benefits Scheme (PBS). The MBS and PBS are the programs through which clinical services and medications, respectively, are subsidized for Australians. Health gains can be measured in a variety of ways. For the proposed study, in addition to clinical outcomes, we will estimate years of life gained and quality-adjusted life years (QALYs) gained. Both will be enabled by the collection of time-to-outcome data and the latter also by collection of the EuroQoL Quality of Life data. All health economic analyses will be undertaken in accordance with recommended approaches, such as 5% discounting of estimated future costs and health gains. To account for any uncertainty in the data inputs for health economic modelling, sensitivity and uncertainty analyses will be undertaken using Monte Carlo simulation techniques [56].

### Project governance

Authors CMS and MM are responsible for overseeing all aspects of the project. Project management support is provided by JW, and additional project management was provided by Neurosciences Trials Australia during the setup phase. The project will be overseen by the project management committee, which consists of CMS, MM, JMcG, CSz, BW, DL, KH, MW, JW and JB and a community representative. This committee meets on a monthly basis. Funding agreements will be entered into between the administering institution and all collaborating institutions. An independent Data Safety Monitoring Committee, comprising a geriatrician and a physiotherapist, both with significant clinical research expertise, will monitor the trial.

### Dissemination policy

The results of the trial will be presented in international peer-reviewed journals and presented at conferences. Decisions about publications arising from the data set will be ratified by the Management Committee and all named investigators will be eligible to have authorship on publications arising from the trial. Trial staff will also provide workshops to clinicians to assist the translation of findings to clinical practice.

## Discussion

Loss of mobility is a major determinant of the need for inpatient rehabilitation for older adults [57]. However, there is currently little evidence to guide clinicians in the management for older people admitted to rehabilitation. It is hypothesized that optimizing physical activity will lead to improved mobility outcomes for this population. It has been established that programs designed to increase physical activity during inpatient rehabilitation can be delivered to most older patients, but whether increasing physical activity during hospitalization is efficacious or cost-effective has not been tested. This study will assess both the shorter and longer term (6 months following discharge) functional outcomes and healthcare utilization of participants who undergo a program to increase their physical activity in addition to usual rehabilitation compared to those who only receive usual rehabilitation plus social interaction. The results of this study will inform current rehabilitation practice in Australia and worldwide, and will contribute to the development of clinical practice guidelines.

## Trial status

The trial commenced recruitment on 28 January 2014 and is currently open for recruitment. Recruitment will cease when 198 participants have been randomized. It is anticipated this target will be reached by April 2015.

## Abbreviations

ACTRN: Australian New Zealand Clinical Trials Registry
CONSORT: Consolidated Standards of Reporting Trials
DEMMI: De Morton Mobility Index
EQ5D: Euro QoL Health Questionnaire
EQ-VAS: Euro QoL Visual Analog Scale
FIM: Functional Independence Measure
IQR: interquartile range
LOS: length of stay
NHMRC: National Health and Medical Research Council
QALYs: quality-adjusted life years
RCT: randomized controlled trial
TUG: timed up and go
